# The Development and Comparative Evaluation of Rosemary Hydroalcoholic Macerate-Based Dermatocosmetic Preparations: A Study on Antioxidant, Antimicrobial, and Anti-Inflammatory Properties

**DOI:** 10.3390/gels11030149

**Published:** 2025-02-20

**Authors:** Alaa Sahlabgi, Dumitru Lupuliasa, Gabriela Stanciu, Simona Lupșor, Lavinia Lia Vlaia, Ramona Rotariu, Nicoleta Corina Predescu, Cristiana Rădulescu, Radu-Lucian Olteanu, Sorina-Geanina Stănescu, Lucian Hîncu, Magdalena Mititelu

**Affiliations:** 1 Department of Pharmaceutical Technology and Biopharmacy, Faculty of Pharmacy, “Carol Davila” University of Medicine and Pharmacy, 020945 Bucharest, Romania; alaa.sahlabgi@drd.umfcd.ro (A.S.); dumitru.lupuliasa@umfcd.ro (D.L.); 2 Department of Chemistry and Chemical Engineering, Ovidius University of Constanta, 900527 Constanta, Romania; 3 Department of Pharmaceutical Technology, Formulation and Technology of Drug Research Center, “Victor Babeș” University of Medicine and Pharmacy, 300041 Timișoara, Romania; vlaia.lavinia@umft.ro; 4 Wastewater Testing Laboratory, RAJA, South Constanta, 900527 Constanta, Romania; ramonarotariu205@gmail.com; 5 Preclinical Sciences Department, Faculty of Veterinary Medicine of Bucharest, University of Agronomic Sciences and Veterinary Medicine of Bucharest, 050097 Bucharest, Romania; corina.predescu@fmvb.usamv.ro; 6 Faculty of Sciences and Arts, Valahia University of Targoviste, 130004 Targoviste, Romania; cristiana.radulescu@valahia.ro (C.R.); radu.olteanu@valahia.ro (R.-L.O.); 7 Institute of Multidisciplinary Research for Science and Technology, Valahia University of Targoviste, 130004 Targoviste, Romania; geanina.stanescu@icstm.ro; 8 Department of Drug Industry and Pharmaceutical Biotechnologies, Faculty of Pharmacy, University of Medicine and Pharmacy Carol Davila, 020956 Bucharest, Romania; lucian.hincu@umfcd.ro; 9 Department of Clinical Laboratory and Food Safety, Faculty of Pharmacy, “Carol Davila” University of Medicine and Pharmacy, 020956 Bucharest, Romania; magdalena.mititelu@umfcd.ro

**Keywords:** *Rosmarinus officinalis*, total phenolic content, total flavonoid content, mineral profile, antioxidant capacity, antibacterial activity, anti-inflammatory activity, skincare, skincare formulations

## Abstract

This study investigates the development and comparative evaluation of new dermatocosmetic preparations based on hydroalcoholic macerates of rosemary (*Rosmarinus officinalis* L.), focusing on their antioxidant, antimicrobial, and anti-inflammatory properties. For this purpose, rosemary hydroalcoholic macerations were analyzed by evaluating the content of biologically active compounds, determining their antioxidant and antimicrobial capacity. Total polyphenol content (TPC), determined via the Folin–Ciocâlteu method, reached 2155 ± 2.45 mg GAE/100 g fresh weight in the 70% ethanol macerate (RDS2) of rosemary from Dobrogea, significantly exceeding (*p* < 0.05) the values observed in the Bulgarian samples. The highest antioxidant activity (745 ± 2.33 mg GAE/100 g fresh weight) correlated with this extraction. Atomic absorption spectroscopy (AAS) analysis revealed elevated calcium (119.5 mg/kg), zinc, and iron levels in Dobrogean rosemary compared to its Bulgarian counterparts. Antimicrobial assessments demonstrated that the 70% ethanol macerate (RDS2) of Dobrogean rosemary exhibited the strongest inhibitory effects, particularly against *Staphylococcus aureus* (inhibition zone: 11–23 mm), while its activity against *Escherichia coli* was moderate (10–17 mm at 30 µL). *Candida albicans* was also significantly inhibited, with an inhibition zone of 9–20 mm. In contrast, the Bulgarian rosemary macerate (RBS2) exhibited weak inhibition against the tested microorganisms. The higher antimicrobial activity of the RDS2 is likely due to its enriched polyphenolic content, including carnosic acid and rosmarinic acid, which are known for their bioactive properties. These findings highlight Dobrogean rosemary’s superior bioactive properties, supporting its use in formulations with antioxidant and antimicrobial benefits.

## 1. Introduction

Rosemary (*Rosmarinus officinalis* L.) is a well known aromatic plant from the *Lamiaceae* family. It is widely used in gastronomy, medicine, and the cosmetic industry due to its high concentration of bioactive compounds, especially polyphenols and essential oils [1,2,3,4,5,6,7]. These constituents contribute to antioxidant, antimicrobial, and anti-inflammatory activities, which have spurred growing scientific interest in rosemary-based applications, particularly in dermatocosmetic formulations [8,9,10,11,12].

The primary bioactive components of rosemary include carnosic acid, carnosol, rosmarinic acid, and essential oils such as eucalyptol, camphor, and bornyl acetate [4,10,11,12]. These compounds are known to be influenced by several factors, including geographical origin, climatic conditions, and the extraction methods used during processing. Rosemary is naturally found in the Mediterranean region and is cultivated in diverse environments, leading to variations in its phytochemical profile [13].

Advanced methods such as ultrasound-assisted extraction (AUE) and supercritical fluid extraction have been shown to significantly improve the yields of bioactive compounds [11]. However, traditional maceration remains a widely used and cost-effective method for obtaining extracts with bioactive potential, especially for dermatocosmetic applications.

The dermatocosmetics industry has experienced a significant shift towards plant-based formulations, driven by consumer demand for natural, sustainable and multifunctional skincare products [13,14].

In addition, research has consistently shown that *Rosmarinus officinalis* L. also contains a diverse mineral composition, including significant levels of calcium, magnesium, iron, and zinc. These mineral concentrations are influenced by external factors such as soil composition and climate, which play an important role in determining the nutrient profile and biological activity of the plant [14,15,16].

The potential application of rosemary (*Rosmarinus officinalis* L.) in dermatocosmetic formulations is supported by its rich profile of bioactive compounds, which provide a number of benefits for skin health, repair, and protection [17,18,19].

One of the primary drivers of rosemary’s potential in skincare formulations is its strong antioxidant activity. The phenolic compounds found in rosemary, such as rosmarinic acid, effectively neutralize free radicals, which are known to accelerate skin aging through oxidative damage to cellular structures, including collagen and elastin. Incorporating rosemary into dermatocosmetic products may help reduce visible signs of aging—such as fine lines, wrinkles, and hyperpigmentation—by providing a protective barrier against environmental stressors, including UV radiation and pollution. This antioxidant activity also promotes healthier skin barrier function, supporting hydration retention and minimizing transepidermal water loss, which is essential for maintaining skin elasticity and smoothness [20,21].

In addition to its antioxidant properties, rosemary has been shown to exhibit significant anti-inflammatory effects, which are beneficial for addressing skin conditions characterized by inflammation, such as acne, eczema, and rosacea. Compounds like carnosol and carnosic acid reduce the expression of pro-inflammatory cytokines, which can alleviate redness, swelling, and discomfort associated with inflammatory skin conditions. This makes rosemary extracts a valuable ingredient for products designed to calm sensitive or irritated skin, enhance skin clarity, and improve overall texture [22,23].

In addition, rosemary extracts demonstrate strong antimicrobial effects, particularly against common skin pathogens including *Staphylococcus aureus*, *Escherichia coli*, and *Candida albicans*. Studies show that optimized extracts of rosemary, such as those macerated in 70% ethanol, exhibit strong antimicrobial activity, helping to control bacterial growth, reduce acne lesions, and maintain a balanced skin microbiome [24,25].

Beyond its functional benefits, rosemary resonates with consumer interest in natural, sustainable ingredients that provide therapeutic effects without the environmental impact associated with synthetic ingredients. As an easily cultivable herb, rosemary represents a renewable resource that supports eco-friendly production practices. This aligns with trends in dermatocosmetics toward minimal bioactive ingredients that are both effective and safe for long-term use. Furthermore, rosemary’s versatility allows it to be formulated across a range of product types, from serums and creams to cleansers and toners, adding a multifunctional component to skincare lines.

To thoroughly evaluate the bioactive properties of rosemary, a series of established assays were employed. Its antioxidant capacity was assessed using the DPPH radical scavenging assay, a widely recognized method for evaluating free radical inhibition potential. Total phenolic content (TPC) was quantified using the Folin–Ciocâlteu method, a highly regarded technique for determining polyphenol content. The mineral compositions of the rosemary extracts were analyzed through atomic absorption spectrometry (AAS), providing comprehensive insights into their elemental profiles, including key minerals such as calcium and zinc [14,15,16].

Despite rosemary’s established benefits, comparative studies on the influence of regional factors on its bioactive composition remain limited. This study provides updated data on rosemary extracts, addressing gaps related to regional variations, extraction efficiency, and dermatocosmetic applications. By integrating these insights into product development, it supports the optimization of rosemary-based skincare formulations with enhanced efficacy and stability.

This study compares the antioxidant activity, mineral composition, and phytochemical profile of rosemary from two geographically distinct regions: Dobrogea (Romania) and the Bulgarian coastal area, where differences in soil and climate are expected to influence bioactive properties. Additionally, the study examines the effects of maceration with ethanol at 96%, 70%, and 40% to optimize bioactive compound extraction and evaluate the antimicrobial activity of the extracts against Gram-positive and Gram-negative bacteria.

The findings provide valuable insights into the impact of geographical, extraction, and microbial factors on rosemary’s chemical composition and biological activity. Extracts with the highest bioactive compound concentrations were further incorporated into hydrogels and dermatocosmetic creams, demonstrating their potential for skincare applications.

The global dermatocosmetic industry has witnessed remarkable growth in recent years, fueled by a surge in consumer demand for natural and sustainable skincare products. In particular, the appeal of plant-based ingredients has skyrocketed, driven by growing concerns over the potential harmful effects of synthetic chemicals. Rosemary (*Rosmarinus officinalis* L.), with its rich bioactive profile, fits seamlessly into this trend. As consumers increasingly seek multifunctional, eco-friendly, and effective products, incorporating rosemary into dermatocosmetics offers both therapeutic benefits and sustainability, addressing consumer preferences for clean beauty products that support skin health and the environment. Rosemary’s demonstrated antioxidant, anti-inflammatory, and antimicrobial properties make it an ideal candidate for addressing a wide range of skin concerns, including aging, acne, eczema, and sensitive skin conditions. This positions rosemary extracts as valuable ingredients in anti-aging creams, acne treatments, and soothing skincare formulations.

As the market for natural skincare expands, formulations leveraging rosemary’s bioactive properties are poised to capture a significant share, especially as consumers become more discerning about ingredient sourcing, quality, and effectiveness. Rosemary’s appeal also stems from its affordability and renewable sourcing, aligning with the increasing demand for eco-conscious production practices in the cosmetic industry.

Despite rosemary’s established potential, comparative studies focusing on the regional variations in its bioactive composition, as well as the impact of extraction methods on its potency, remain sparse. Current research predominantly focuses on broad applications of rosemary, yet there is a lack of comprehensive studies examining the geographical influence on its chemical profile and biological activity, especially concerning dermatocosmetic formulations. The Dobrogea region in Romania, with its unique climatic and soil conditions, has not been thoroughly studied in the context of rosemary’s potential for skincare applications, especially in comparison with Bulgarian rosemary samples.

There is a clear research gap in understanding how different extraction solvents, such as ethanol concentrations, affect the antioxidant, antimicrobial, and anti-inflammatory properties of rosemary and how these properties influence the formulation of effective dermatocosmetics. The influence of mineral composition on rosemary’s biological activity also remains underexplored, despite minerals like calcium, zinc, and iron potentially enhancing skin health and contributing to the efficacy of rosemary-based skincare products.

## 2. Results and Discussion

### 2.1. Total Phenolic Content (TPC) and Antioxidant Activity of Rosemary Hydroalcoholic Macerates for Dermatocosmetic Applications

**Figure 1:**
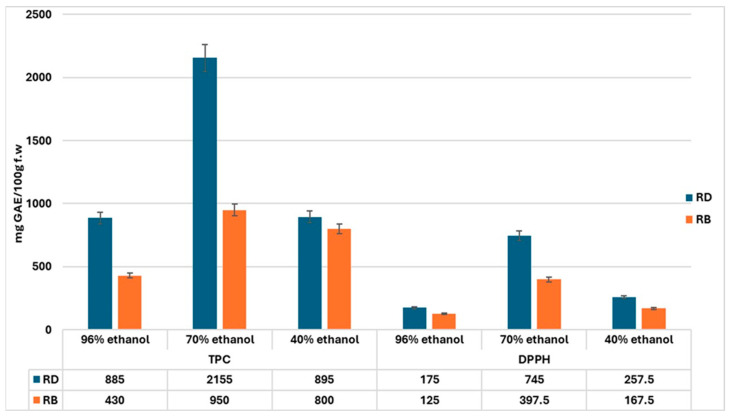
Total phenolic content (TPC) and antioxidant activity evaluated by DPPH assay in *Rosmarinus officinalis* samples from Dobrogea (RD) and Bulgaria (RB).

The total phenolic content (TPC) and antioxidant activity of *Rosmarinus officinalis* hydroalcoholic macerates were assessed to determine their potential for use in dermatocosmetic formulations. The results, expressed in mg of gallic acid equivalents per 100 g of fresh material (mg GAE/100 g f.w.), are presented in Figure 1.

The results substantiate the pivotal role of solvent polarity in phenolic compound extraction, demonstrating the superior efficacy of 70% ethanol. Significant variations in total phenolic content (TPC) and antioxidant activity were observed across the samples.

The highest TPC was recorded in Dobrogean rosemary macerated with 70% ethanol (RDS2), reaching 2155 ± 2.45 mg GAE/100 g f.w., while the lowest was found in Bulgarian rosemary extracted with 96% ethanol (RBS1) (430 ± 1.25 mg GAE/100 g f.w.). These findings underscore the preferential solubilization of phenolic compounds in 70% ethanol, attributed to its balanced polarity, which facilitates the extraction of both hydrophilic and lipophilic phenolics (Figure 1).

The data suggest that solvent polarity plays a critical role in the efficiency of total phenolic extraction. Ethanol at a 70% concentration (Solvent 2) consistently provided higher extraction yields compared to 96% (Solvent 1) or 40% ethanol (Solvent 3), likely due to its balanced polarity, which enhances the solubility of both hydrophilic and lipophilic phenolic compounds. This trend is particularly evident in the Dobrogean rosemary samples, where the 70% ethanol extract (RDS2) showed a markedly higher TPC than the other solvent concentrations.

A key observation in this study is the significant difference in TPC between the Dobrogean and Bulgarian rosemary samples. This disparity may be attributed to several regional factors such as climate, soil composition, and agricultural practices, which can influence the accumulation of phenolic compounds in *Rosmarinus officinalis*. The Dobrogean rosemary samples consistently demonstrated higher TPCs, regardless of ethanol concentration, than their Bulgarian counterparts.

To rigorously assess the statistical significance of these regional differences, we conducted an independent *t*-test to compare the TPCs between the two regions. The results showed a highly significant difference (*p* < 0.01) in the phenolic content between Dobrogean and Bulgarian rosemary, with Dobrogean extracts having consistently higher phenolic concentrations across all solvent concentrations.

Further statistical analysis using a two-way ANOVA was performed to examine the combined effects of ethanol concentration and geographical origin on TPC. The interaction term was found to be significant (*p* < 0.001), indicating that both factors influence the total phenolic yield in rosemary extracts, but the effect of geographical origin was more pronounced. A post hoc Tukey’s HSD test further confirmed that the rosemary from Dobrogea consistently exhibited higher TPC across all ethanol concentrations compared to the Bulgarian samples.

A parallel trend was observed in antioxidant activity, assessed via DPPH assay. The Dobrogean rosemary macerate (RDS2) exhibited the highest radical scavenging activity (745.0 ± 2.33 mg GAE/100 g f.w.), while its Bulgarian counterpart (RBS2) demonstrated significantly lower activity (397.5 ± 1.76 mg GAE/100 g f.w.).

Beyond solvent efficiency, the observed disparities between the Dobrogean and Bulgarian samples suggest an additional influence of geographical factors, including climatic conditions, soil composition, and agricultural practices, on phenolic biosynthesis in *Rosmarinus officinalis*. These results emphasize the interplay between extraction parameters and plant origin, offering critical insights for optimizing bioactive compound recovery in dermatocosmetic formulations and functional food applications.

These findings align with existing literature, supporting the enhanced solubility of phenolics in hydroalcoholic solutions within the 50–70% ethanol range [1,2,3,4,5,13]. Notably, the TPC values obtained exceed those reported in comparable studies, where rosemary extracts typically range between 1500 and 2000 mg GAE/100 g, contingent on geographical origin and extraction methodology [2]. The consistently higher polyphenol content and antioxidant activity in Dobrogean rosemary suggest that the environmental conditions in this region favor bioactive compound biosynthesis.

Overall, this study underscores the synergistic influence of solvent selection and geographical origin in determining the phenolic profile and antioxidant potential of rosemary extracts, offering a foundation for optimized extraction strategies in pharmaceutical, nutraceutical, and dermatocosmetic applications.

### 2.2. Total Flavonoid Content (TFC) Activity of Rosemary Hydroalcoholic Macerates for Dermatocosmetic Applications

**Figure 2:**
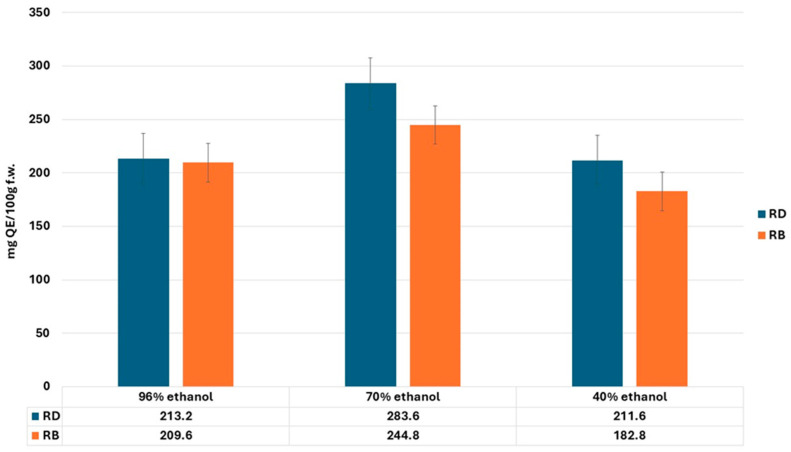
Total Flavonoid Content evaluation of *Rosmarinus officinalis* samples from Dobrogea (RD) and Bulgaria (RB).

The Total Flavonoid Content (TFC) in the *Rosmarinus officinalis* samples from Dobrogea and Bulgaria revealed significant differences (Figure 2). The highest TFC value was observed also in the Dobrogean sample macerated with 70% ethanol (RDS2), with a value of 283.6 ± 1.52 mg QE/100 g fresh weight (f.w.), which strongly correlates with its superior total phenolic content and antioxidant activity. This suggests that the 70% ethanol extraction efficiently solubilizes flavonoids, which play a pivotal role in enhancing the radical scavenging potential of rosemary.

The Bulgarian rosemary samples generally showed lower TFCs compared to their Dobrogean counterparts, the highest TFC among them being 244.8 ± 1.65 mg QE/100 g f.w. in RBS2 (70% ethanol). The lower values of TFC in the Bulgarian samples can be attributed to regional factors such as soil composition and climatic conditions.

A good correlation was found between TFC and antioxidant activity, as demonstrated by the DPPH assay. Samples with higher TFCs, such as RDS2 and RBS2, showed stronger radical scavenging activity, confirming the direct role of flavonoids in increasing the biological efficacy of rosemary.

The higher TFC values observed in the samples from Dobrogea indicate that rosemary from this region is a richer source of flavonoids and recommend exploiting its therapeutic potential, especially for obtaining new dermatocosmetic preparations effective in managing oxidative stress on the skin, reducing signs of aging, and protecting against microbial imbalance, which can lead to acne and irritation [19].

The results of the Pearson correlation analysis highlight significant and strongly positive associations between the studied variables. The correlation between TPC and AA is very strong (r = 0.953, *p* = 0.003), indicating a close link between them. The relationship between TPC and TFC also presents a significant positive correlation (r = 0.842, *p* = 0.035), and an extremely strong correlation was identified between AA and TFC (r = 0.937, *p* = 0.006). All correlations are statistically significant at the 95% confidence level (*p* < 0.05), suggesting that the observed associations are not due to chance (Table 1).

**Figure 3:**
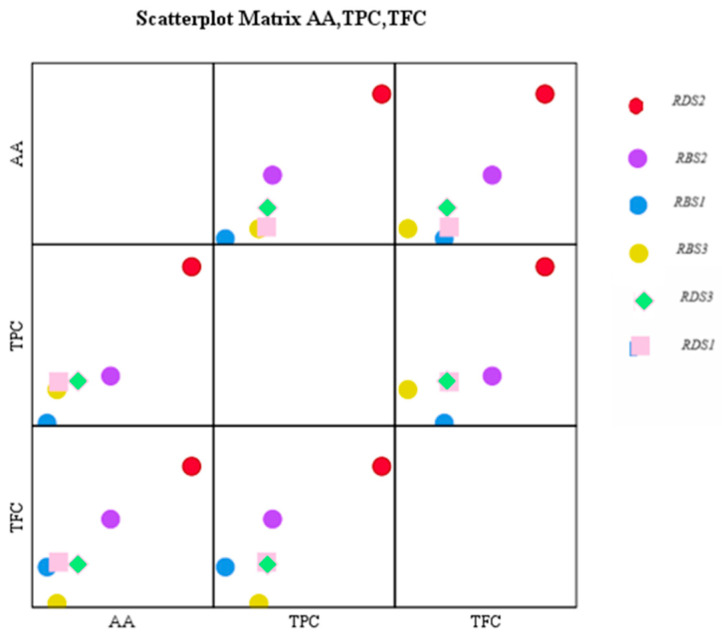
The correlation matrix of the AA, TPC, and TFC of the rosemary extracts.

A scatterplot analysis between the AA, TPC, and TFC variables (Figure 3) indicates a positive correlation between antioxidant activity and total phenolic compounds content (TPC), which is consistent with the literature, with phenols being recognized for their antioxidant properties. The relationship between TFC and AA, although present, is weaker, suggesting that not all flavonoids contribute to antioxidant activity directly. The strong correlation between TPC and TFC validates that flavonoids constitute an important proportion of total phenolic compounds.

After analyzing the data using Student’s *t*-test for one sample, statistically significant results were obtained for all three analyzed variables (TPC, AAE, and TFC) (Table 2). The t-statistic value and the significance level (*p*-value) indicate the existence of significant differences from the reference value (0). More specifically, TPC records a mean difference of 1019.17 (t(5) = 4.252, *p* = 0.008), with a 95% confidence interval [403.04, 1635.30], which confirms a significant difference. AAE also presents a mean difference of 311.25 (t(5) = 3.266, *p* = 0.022), and the 95% confidence interval [66.31, 556.19] indicates that the mean is significantly different from the reference value. The strongest difference is observed in the case of TFC, where the mean difference is 224.27 (t(5) = 15.651, *p* < 0.001), and the 95% confidence interval [187.43, 261.10] supports the robustness of this result.

**Figure 4:**
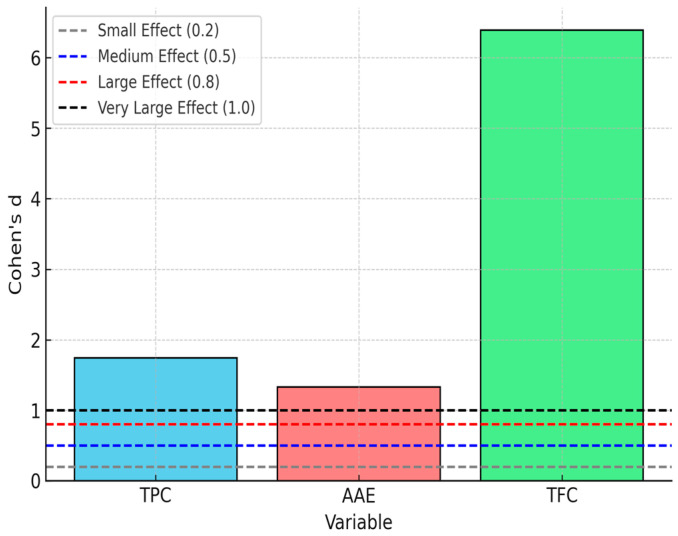
Effect size (Cohen’s d) for each variable.

To determine the adequacy of the sample size, we conducted both an a priori and a post hoc power analysis. Using Cohen’s d effect size for each variable, the calculated values were d = 1.74 for TPC, d = 1.33 for AAE, and d = 6.39 for TFC, all of which indicate very large effects (Figure 4). Given these values, the post hoc statistical power of our study significantly exceeds the conventional threshold of 0.80, ensuring a high probability of detecting true effects. Additionally, our a priori power analysis confirmed that the current sample size was sufficient for robust statistical inference. These findings suggest that our sample size was appropriate for detecting significant effects, reinforcing the reliability of our conclusions.

### 2.3. Mineral Content of Rosemary Hydroalcoholic Macerates for Dermatocosmetic Applications

**Figure 5:**
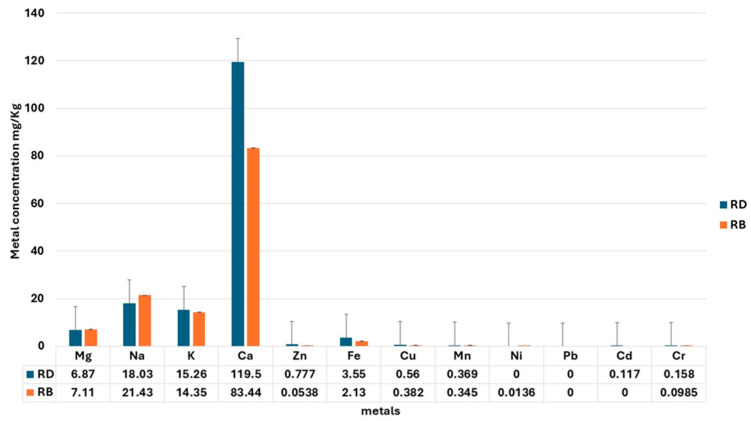
Mineral content of *Rosmarinus officinalis* fresh leaf samples from Dobrogea (RD) and Bulgaria (RB) (mg/kg f.w.).

The analysis of the mineral content in the rosemary samples from Dobrogea (RD) and Bulgaria (RB) revealed notable differences in key elements, as seen in Figure 5.

The rosemary samples from Dobrogea (RD) consistently demonstrated higher concentrations of essential minerals compared to the Bulgarian samples (RB), which could be attributed to regional environmental factors such as soil composition and climate.

The highest concentration was observed for Ca, with the RD samples containing 119.5 mg/kg, significantly higher than the 83.44 mg/kg found in the RB samples. Calcium is essential for both plant structural integrity and human health, and the higher content in Dobrogean rosemary may indicate a better nutritional profile. The sodium and potassium levels were fairly similar between the regions, with RD showing slightly lower Na (18.03 mg/kg) but higher K (15.26 mg/kg) values compared to RB, which had a Na value of 21.43 mg/kg and a K of 14.35 mg/kg. These minerals are essential for maintaining cellular function in both plants and humans. Magnesium concentrations were comparable between the regions, with RD at 6.87 mg/kg and RB at 7.11 mg/kg.

Trace elements, including Zn and Fe, were present in higher concentrations in the RD samples. Zinc, an essential element for enzyme function and protein synthesis, was measured at 0.777 mg/kg in RD, substantially higher than the 0.0538 mg/kg found in RB. Similarly, Fe, critical for oxygen transport in plants and humans, was higher in RD (3.55 mg/kg) compared to RB (2.13 mg/kg). Copper (Cu) and Mn concentrations were also slightly elevated in the RD samples, with Cu at 0.56 mg/kg and Mn at 0.369 mg/kg, compared to 0.382 mg/kg and 0.345 mg/kg, respectively, in RB. Heavy metals, including Pb, Ni, Cd, and Cr, were detected at very low levels in both regions, well below safe thresholds for human consumption. This indicates that rosemary from both regions is safe for use in food and nutraceutical applications.

By fortifying dermatocosmetic formulations with rosemary-derived minerals, manufacturers can create products that not only protect but also actively support skin renewal and balance [26,27,28].

### 2.4. Antimicrobial Activity of Rosemary Hydroalcoholic Macerates for Dermatocosmetic Applications

The antimicrobial activity results are summarized in Table 3. The antimicrobial activity of the rosemary macerates RDS2 and RBS2 was evaluated against three microorganisms: *Escherichia coli* ATCC 25922, *Staphylococcus aureus* ATCC 25923, and *Candida albicans* ATCC 10231. The results revealed a differential response between Gram-positive and Gram-negative bacteria, as well as yeast, based on the type of macerate and the volume tested.

The results confirm that the RDS2 macerate exhibits superior antimicrobial activity compared to RBS2, particularly against Gram-positive bacteria and yeast. This difference may be attributed to the higher content of polyphenolic compounds, such as carnosic acid and rosmarinic acid, in RDS2, which are known for their potent antimicrobial properties.

*Staphylococcus aureus* demonstrated greater sensitivity to both extracts compared to *Escherichia coli*, which aligns with the general observation that Gram-negative bacteria possess a complex outer membrane rich in lipopolysaccharides, limiting the permeability of hydrophobic antimicrobial compounds and reducing the effectiveness of plant-derived antimicrobials.

The results demonstrate that RDS2 has significant antimicrobial potential, particularly against Gram-positive bacteria and *Candida albicans*, while its effectiveness against Gram-negative bacteria is moderate. RBS2, despite being derived from the same plant, showed weaker antimicrobial activity across all tested organisms.

The data suggest that the polyphenolic profile of the rosemary extracts plays an important role in determining their antimicrobial efficacy, with RDS2 being richer in bioactive compounds responsible for these effects.

Studies have shown that rosemary extracts can inhibit Staphylococcus aureus and *Escherichia coli*, common culprits in skin infections and imbalances. This antimicrobial action is believed to be due to the ability of rosemary compounds to disrupt microbial cell walls, interfere with protein synthesis, and induce oxidative stress in microbial cells [11,17]. Additionally, rosemary’s effects on fungal species such as *Candida albicans* suggest that it may be beneficial for managing skin conditions related to fungal overgrowth. This growing body of evidence supports the use of rosemary in skincare formulations and topical treatments aimed at reducing pathogen load and supporting a healthy skin microbiome [29].

For instance, in one study, rosemary extract demonstrated strong inhibitory effects on *Staphylococcus aureus*, with a minimum inhibitory concentration (MIC) of 2 mg/mL, highlighting its potential to combat Gram-positive bacteria often implicated in skin infections and acne. Similarly, for *Escherichia coli*, a common Gram-negative bacterium, rosemary extracts showed MIC values ranging from 2 to 4 mg/mL depending on the extraction method and concentration. This suggests that rosemary could be an effective natural alternative to synthetic antimicrobials, particularly for products aimed at managing oily and acne-prone skin [30].

The antimicrobial efficacy of rosemary macerates varies depending on the concentration of the hydroalcoholic solvent used. For example, a 70% ethanol extract of rosemary inhibited the growth of *S. aureus* by 85% at a concentration of 3 mg/mL, compared to only 40% inhibition by a 40% ethanol extract at the same concentration. For *Escherichia coli*, the minimum inhibitory concentration (MIC) values ranged from 2 to 4 mg/mL, while *Candida albicans* showed an MIC of 0.5 mg/mL [20,31,32].

These findings underscore rosemary’s broad-spectrum antimicrobial properties, supporting its use as a natural, multifunctional ingredient in dermatocosmetic formulations designed to address bacterial and fungal concerns.

### 2.5. Characteristics of Dermatocosmetic Preparations Based on Rosemary Hydroalcoholic Macerates

**Figure 6:**
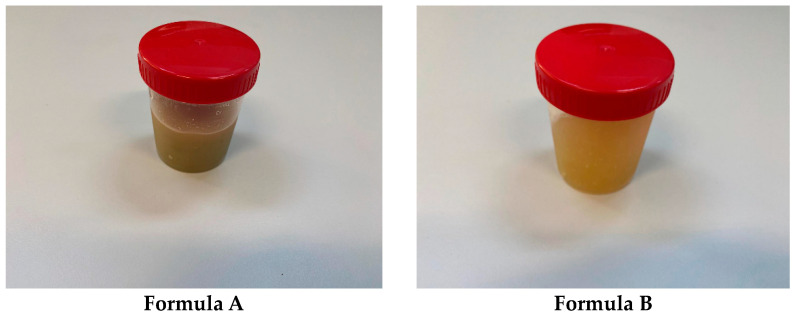
Hydrogels based on rosemary macerates.

The characteristics of the analyzed hydrogels are presented in Table 4 and Figure 6.

**Figure 7:**
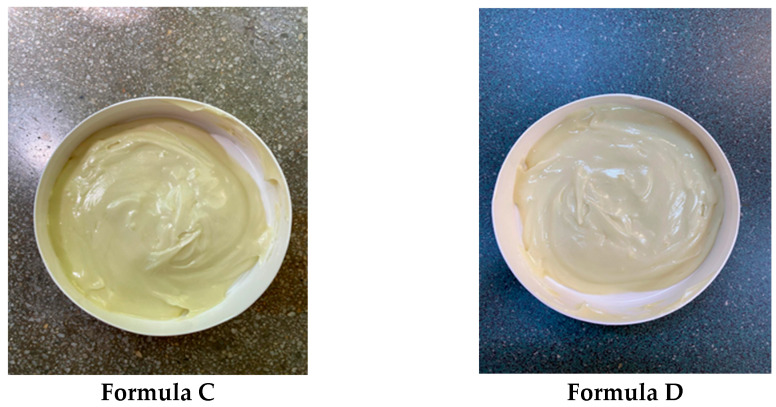
Dermatocosmetic creams based on rosemary macerates.

The characteristics of the analyzed dermatocosmetic creams are presented in Table 5 and Figure 7.

From a macroscopic perspective, all four dermatocosmetic preparations analyzed exhibit homogeneity, ensuring the uniform distribution of active ingredients throughout the formulations. The stability of these preparations is noteworthy, as no phase separation, sedimentation, or visible changes in texture were observed over time, highlighting their robustness under standard storage conditions. Additionally, their pH values are compatible with the physiological pH of the skin, minimizing the risk of irritation and making them suitable for long-term dermal application. In dermatocosmetic formulations, maintaining a pH within the skin’s natural range (4.5–5.5) is important to avoid irritation, the disruption of the skin barrier, or interference with the skin’s natural microbiota. Products with a pH outside this range—especially those that are too alkaline (e.g., >7)—can strip natural oils, increase dryness, and potentially harm the skin barrier. Conversely, products that are overly acidic (e.g., <3) might cause irritation or sensitivity, particularly in damaged or sensitive skin types [33]. Furthermore, the viscosity of the formulations demonstrated minimal variation over time, indicating consistent rheological behavior and optimal spreadability, which are essential for maintaining ease of application and user satisfaction. According to the results presented in Table 4 and Table 5, it is observed that the organoleptic characteristics remain unchanged over time (after 30 and 60 days from preparation) and there are only very small variations in terms of pH and viscosity.

**Figure 8:**
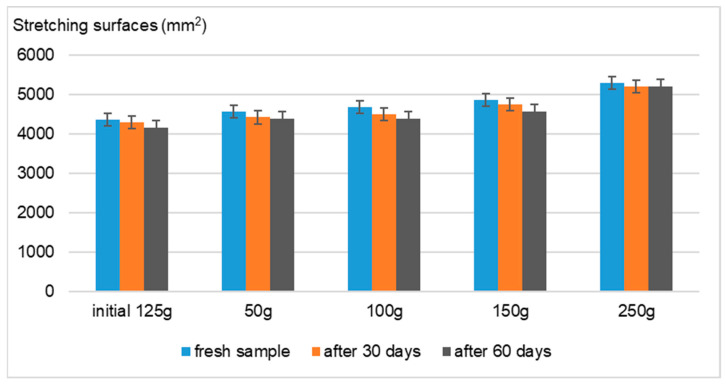
Spreadability of Formula A.

**Figure 9:**
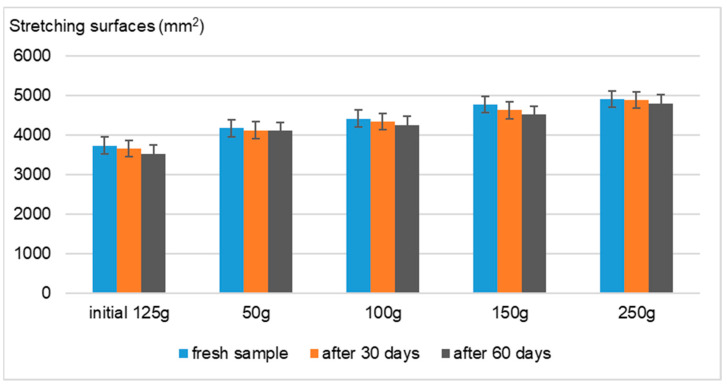
Spreadability of Formula B.

**Figure 10:**
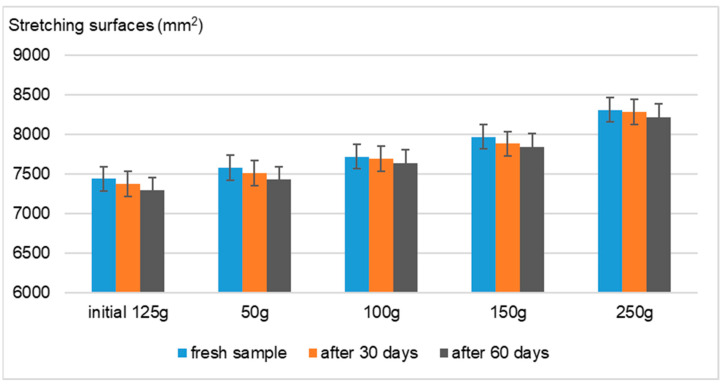
Spreadability of Formula C.

**Figure 11:**
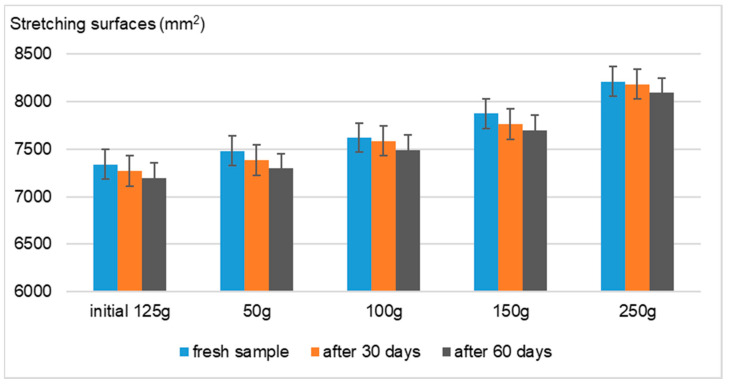
Spreadability of Formula D.

Using the Ojeda–Arboussa method for analysis, the graphs presented in Figure 8, Figure 9, Figure 10 and Figure 11 illustrate a high degree of spreadability for the dermatocosmetic preparations evaluated. The data reveal that the two hydrogels and the two cosmetic creams exhibit comparable spreadability, with only minor differences observed between formulations within the same category. Furthermore, the spreadability parameters remained stable over time, indicating the formulations’ consistency and suitability for prolonged use. These findings suggest that the physical characteristics of the preparations, such as viscosity and texture, are well-balanced to ensure effective application and user satisfaction [34].

**Figure 12:**
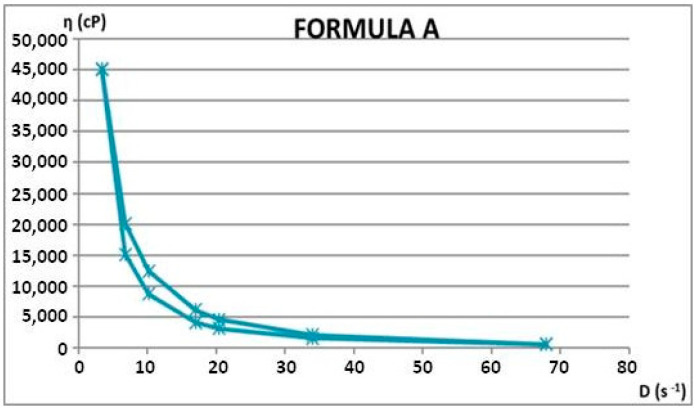
Flow curve for Formula A.

**Figure 13:**
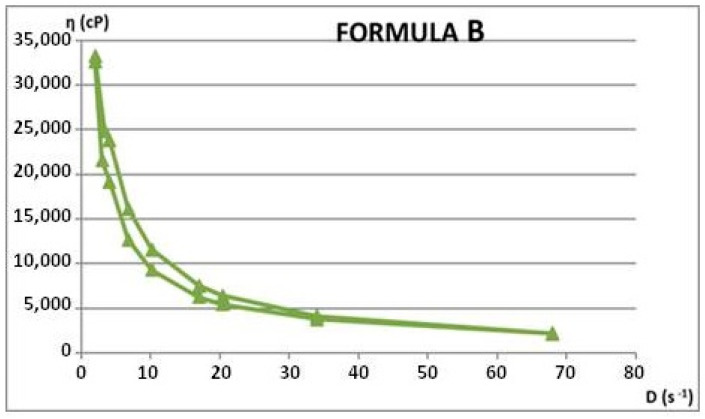
Flow curve for Formula B.

**Figure 14:**
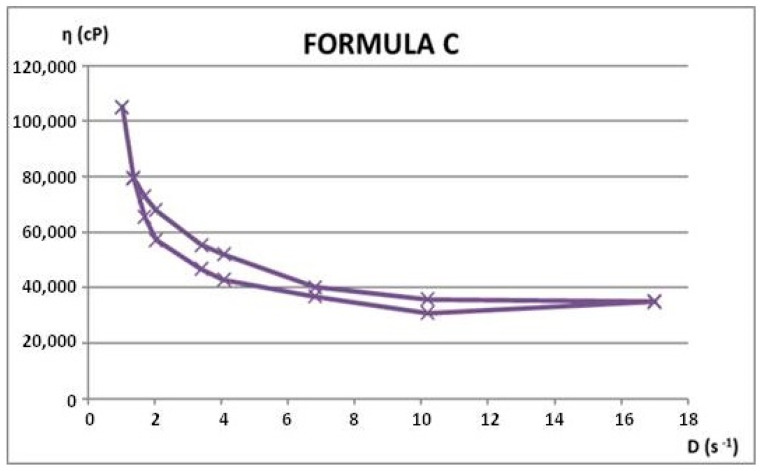
Flow curve for Formula C.

**Figure 15:**
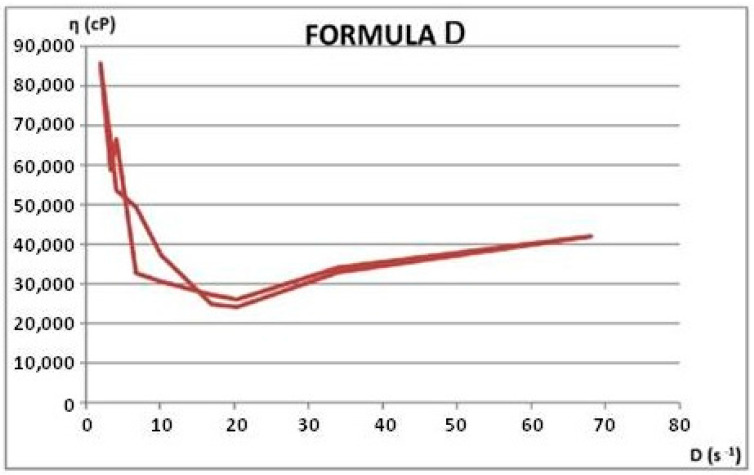
Flow curve for Formula D.

Rheological properties were assessed through viscosity measurements at varying rotational speeds, both ascending and descending. From these measurements, shear rate and shear stress values were calculated. Using the average rheological parameters, rheograms and flow curves were generated to illustrate the materials’ flow behavior. Apparent viscosity (η, in cP) readings were taken in triplicate for each rotational speed (ω, in rpm), during both the increasing and decreasing phases. The rheological attributes of the dermatocosmetic formulations are detailed in Table 6 and Table 7 and visualized in Figure 12, Figure 13, Figure 14 and Figure 15.

The rheological attributes of the Formula A and Formula B hydrogels, developed using a hydroalcoholic extract of rosemary, demonstrate the key characteristics of well-structured formulations. Both formulas exhibited favorable viscosity profiles, ensuring ease of application and stable adherence to the skin’s surface.

For Formula A, the rheological behavior reflected a pseudoplastic nature, with a decrease in viscosity under shear stress, facilitating smooth application without dripping. The recovery of viscosity after the removal of shear stress highlighted its structural integrity, essential for maintaining product stability during storage and usage.

Formula B demonstrated similar pseudoplastic behavior, with slightly higher viscosity values, indicating enhanced skin adhesion and a controlled release profile for the active compounds. Both formulas maintained consistent rheological properties across varying temperatures, ensuring robustness and performance under diverse conditions.

These characteristics underscore the hydrogels’ suitability for dermatocosmetic applications, combining ease of use, stability, and the effective delivery of rosemary extract’s therapeutic benefits.

The rheological properties of the Formula C and Formula D dermatocosmetic creams, formulated with a hydroalcoholic extract of rosemary, exhibit essential attributes for effective and user-friendly applications.

Formula C demonstrates an optimal balance of viscosity and shear-thinning behavior, characteristic of a pseudoplastic system. This ensures that the cream spreads smoothly on the skin under the application’s mechanical force while regaining its viscosity afterward, aiding in product stability and prolonged skin adherence. Its consistency supports uniform application and efficient delivery of active rosemary compounds.

Formula D shows a slightly thicker texture with enhanced viscosity, designed for prolonged skin contact and a controlled release of the bioactive components. Its rheological profile indicates strong structural integrity, making it particularly suitable for targeted or prolonged treatments. The cream’s ability to retain its rheological properties under different storage conditions reinforces its reliability and effectiveness.

Both formulations possess desirable spreadability, stability, and adaptability, making them highly suitable for dermatocosmetic use. Their rheological performance supports the effective delivery of rosemary extract’s antioxidant and soothing properties, aligning with user expectations for high-quality skincare products.

The analysis of the characteristics of the dermatocosmetic preparations developed based on hydroalcoholic extracts of rosemary demonstrates the efficiency of preformulation tests that allow the creation of stable preparations with good rheological characteristics.

In the regression analysis, the dependent variable, pH, was modeled according to the predictor “Viscosity”. The model presents a standard error of estimation of 0.28881, which reflects the average deviation of the observed values of the pH variable from those estimated by the model. The coefficient of determination, R^2^ = 0.102, means that 10.20% of the variance of the dependent variable “pH” can be explained by the variance of the independent variable (Viscosity). In Table 8, the unstandardized (B) and standardized (beta) regression coefficients are presented, as well as the results of the *t*-tests for each of these coefficients. For the simple linear regression, beta (standardized coefficient β = 0.320) is the correlation coefficient between the independent variable and the dependent variable. The unstandardized coefficient for the ethical attitudes and political level variable is b = 0.001 and represents the slope of the regression line. The unstandardized coefficient for the constant is a = 4.736, which represents the intercept.

The regression model (Table 9) that expresses the viscosity as a function pH is described by Equation Y: a + bX = −4.736 + 0.001X.

**Figure 16:**
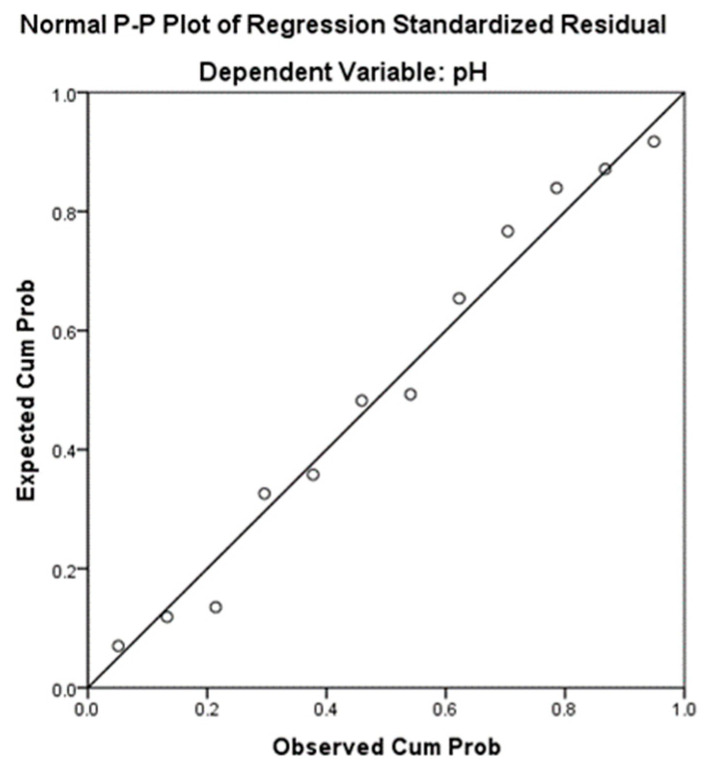
Normal P-P plot of regression-standardized residual.

On the other hand, to analyze the relationship between pH and viscosity, the scatter plot in Figure 16 indicates that there is a moderate positive linear relationship between the two variables, which is supported by the correlation coefficient (r = 0.320).

### 2.6. Antioxidant Capacity of Dermatocosmetic Preparations

The total antioxidant capacity of the encoded of dermatocosmetic preparations, compared to the Trolox^®^ standard, was quantified according to the ACL (Analytik Jena AG, Jena, Germany) procedure for the stock solutions and for 1:100 diluted stock solutions. The quantitative results, expressed in mg TE/100 g sample, are shown in Table 10.

According to the results presented in Table 10, the highest antioxidant activities were shown by Formula C followed by Formula A.

In dermatocosmetic studies, rosemary extracts have shown high efficacy in neutralizing free radicals, supporting skin barrier function, and promoting skin clarity, making them especially suitable for anti-aging and calming formulations [17]. Additionally, the antimicrobial activity of rosemary against pathogens like *Staphylococcus aureus* and *Escherichia coli* positions it as a natural ingredient for products targeting acne-prone skin. Hydroalcoholic extractions at 70% ethanol have been found to optimize these bioactive properties, enhancing rosemary’s solubility and efficacy in formulations. This research collectively highlights rosemary as a versatile, plant-based ingredient for dermatocosmetic products focused on skin health and resilience [19].

### 2.7. Anti-Inflammatory Activity Results

**Figure 17:**
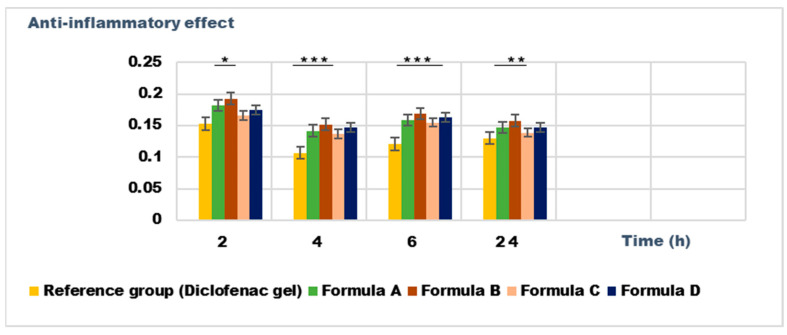
The anti-inflammatory effects on inflammatory edema induced with 10% kaolin suspension. The data are represented as means ± SD and analyzed using ANOVA. * *p* < 0.05, ** *p* < 0.01, and *** *p* < 0.001 versus the control group.

**Figure 18:**
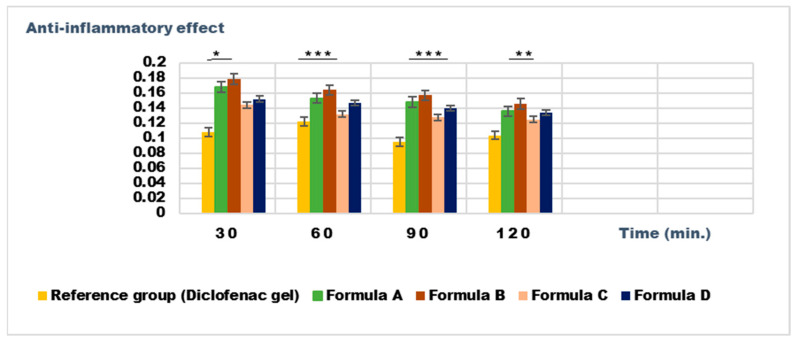
The anti-inflammatory effects on inflammatory edema induced with a 6% dextran solution. The data are represented as means ± SD and analyzed using ANOVA. * *p* < 0.05, ** *p* < 0.01, and *** *p* < 0.001 versus the control group.

The experimental results of the evaluations of anti-inflammatory effects carried out on Formulas A, B, C, and D with rosemary macerated in 70% ethanol are presented in Figure 17 and Figure 18.

The experimental results demonstrate a significantly greater anti-inflammatory effect for dermatocosmetic creams compared to hydrogels, as evidenced by their ability to substantially reduce inflammation levels post-treatment. This enhanced effect can be attributed to the synergistic action of rosemary macerates in 70% ethanol and the additional components in the cream formulations, particularly the ingredients in the fat phase, which aid in improving the skin penetration and bioavailability of the active compounds [35].

Among all the formulations tested, Formula C exhibited the most pronounced anti-inflammatory activity. This was particularly evident in the reduction in kaolin-induced edema at 4 h post-induction (Figure 17) and in the suppression of dextran-induced edema at 90 min post-inflammation induction (Figure 18). The edema inhibition (E%) for inflammation induced by dextran solution is 49.2% for Formula C after 90 min and 62.3% for the reference group, and in the case of inflammations induced by kaolin suspension it is 49.63% for Formula C after 4 h and 60.66% for the reference group, the data indicating a significant anti-inflammatory effect for Formula C.

Furthermore, the results indicate that dermatocosmetic preparations containing rosemary macerates sourced from the Dobrogea region displayed significantly stronger anti-inflammatory effects compared to those formulated with extracts from the Bulgarian region. This disparity is attributed to differences in the chemical composition of the rosemary extracts, with Dobrogean macerates showing higher levels of bioactive compounds, such as phenolics and flavonoids, which are known to contribute to anti-inflammatory activity [36,37,38,39].

These findings emphasize the potential of Dobrogean rosemary-based formulations, particularly creams, in enhancing anti-inflammatory properties, making them ideal candidates for therapeutic and dermatocosmetic applications.

The selection of 70% ethanol as the optimal concentration for extracting rosemary macerates is based on its ability to efficiently extract a wide range of bioactive compounds, such as polyphenols, essential oils, and flavonoids, while maintaining antioxidant, antimicrobial, and anti-inflammatory properties. Furthermore, it offers a balanced solvent polarity, cost-effectiveness, safety, and consumer acceptance in dermatocosmetic applications. This makes 70% ethanol the ideal concentration for creating stable, effective, and safe rosemary-based formulations that meet the growing demand for natural, multifunctional skincare products.

70% ethanol is a well-balanced solvent because it offers a high extraction yield while maintaining a good solvent polarity. Ethanol is a polar solvent that can efficiently extract hydrophilic compounds such as polyphenols, flavonoids, and minerals, while also being capable of extracting lipophilic compounds like essential oils.

The choice of 70% ethanol allows for better penetration and extraction efficiency compared to more diluted solvents like 40% ethanol, which may fail to extract sufficient bioactive compounds. Conversely, 96% ethanol, although a stronger solvent, can result in the excessive evaporation of volatile compounds like essential oils, thus reducing their concentration in the final extract.

Rosemary contains significant amounts of polyphenolic compounds such as rosmarinic acid, carnosic acid, and carnosol, which are key to its antioxidant, anti-inflammatory, and antimicrobial properties. Research has shown that a concentration of 70% ethanol is particularly effective for extracting polyphenols, as it balances the solubility of water-soluble compounds (such as polyphenols) and lipophilic compounds (such as essential oils). Rosemary is also known for its bioactive essential oils (e.g., eucalyptol, camphor, and bornyl acetate). At 70% ethanol, the solvent is effective in extracting a good balance of volatile compounds from rosemary, ensuring that both polar and non-polar bioactive compounds are captured. Higher ethanol concentrations (e.g., 96%) can sometimes result in the incomplete extraction of essential oils, while lower ethanol concentrations (e.g., 40%) may not extract enough of the polyphenolic compounds.

Rosemary (*Rosmarinus officinalis* L.) provides versatile, practical applications in the dermatocosmetic market, offering natural solutions for a wide range of skin concerns, from anti-aging to acne, sensitive skin, hydration, and sun protection. As consumer demand for natural, sustainable, and multifunctional skincare products continues to rise, rosemary-based formulations stand out as effective, safe, and eco-friendly alternatives in the crowded skincare market. By harnessing its bioactive properties and bioavailability, rosemary offers a rich opportunity for formulators to develop innovative, high-performance dermatocosmetic products that meet the evolving needs of consumers.

Study Limitations:

This study focuses on maceration with ethanol at concentrations of 96%, 70%, and 40%, with no evaluation of other advanced extraction methods (e.g., ultrasound-assisted extraction (AUE) or supercritical fluid extraction (SFE)). While maceration is a widely used and cost-effective method, further comparisons with these advanced techniques could reveal more efficient ways to extract bioactive compounds, especially for scalable dermatocosmetic production.

The study does not fully address the potential interactions between rosemary extracts and other common ingredients used in dermatocosmetic formulations (e.g., preservatives, emulsifiers, humectants). These interactions could influence the stability, texture, and performance of the final products. Further research is required to understand how rosemary’s bioactive compounds interact with different formulation components.

Further investigation is needed to optimize extraction methods, assess regional variations in rosemary’s bioactive profile, and validate its potential in dermatocosmetic formulations, particularly hydrogels and creams designed to address various skin concerns. Addressing these gaps will not only enhance the effectiveness and stability of rosemary-based products but also contribute to the broader knowledge of plant-based ingredients in skincare, meeting the growing demand for scientifically supported and sustainably sourced dermatocosmetic solutions.

## 3. Conclusions

This study highlights the development and comparative evaluation of dermatocosmetic preparations formulated with hydroalcoholic macerates of *Rosmarinus officinalis* from Dobrogea (Romania) and Bulgaria, with a focus on their antioxidant, antimicrobial, and anti-inflammatory properties. The findings demonstrate that the 70% ethanol macerate from Dobrogea (RDS2) contained the highest total phenolic and flavonoid contents, which correlated with superior antioxidant capacity.

Regarding antimicrobial activity, RDS2 exhibited notable efficacy against Gram-positive bacteria (*Staphylococcus aureus*) and yeast (*Candida albicans*), while its activity against Gram-negative bacteria (*Escherichia coli*) was moderate. In contrast, the Bulgarian rosemary macerate (RBS2) demonstrated limited antimicrobial effects, with inhibition only at higher concentrations.

The dermatocosmetic formulations developed in this study—including hydrogels and creams incorporating rosemary macerates—were physicochemically stable and exhibited notable antioxidant and anti-inflammatory effects. The cream formulation containing RDS2 (Formula C) displayed the most pronounced anti-inflammatory activity, particularly in kaolin- and dextran-induced inflammation models, confirming its potential for soothing and therapeutic dermatological applications.

These findings underscore the importance of optimizing extraction methods and selecting high-quality botanical sources to enhance the efficacy of rosemary-based dermatocosmetic products. Further studies should focus on improving the antimicrobial potential against Gram-negative bacteria and fungi, refining formulation stability, and expanding the scope of applications in skincare, pharmaceuticals, and nutraceuticals.

## 4. Materials and Methods

Reagents

All reagents used for chemical determinations were of analytical reagent grade. Gallic acid was sourced from Fluka (Buchs, Switzerland), and Folin–Ciocâlteu reagent was obtained from Merck (Darmstadt, Germany). A standard solution of gallic acid (34 µg/mL) was prepared and the Folin–Ciocâlteu reagent was diluted in distilled water at a ratio of 1:2 (*v*:*v*). DPPH (2,2-diphenyl-1-picrylhydrazyl) was prepared as a 0.0063% (1.268 mM) standard solution in methanol. Quercetin was prepared as a standard solution (10 mg/100 mL) in methanol.

Spectrophotometric analyses were performed using a JASCO V550 UV-VIS scanning spectrophotometer (JASCO, Tokyo, Japan) for determining the total phenolic content via the Folin–Ciocâlteu method and for evaluating antioxidant activity using the DPPH radical scavenging assay.

Sample collection and preparation

For this study, fresh leaves of *Rosmarinus officinalis* L. were collected in June 2023 from two specific locations: the Dobrogea Region, Romania, near Constanța (44°10′ N, 28°38′ E), from a semi-wild habitat in the coastal area, and the Bulgarian coastal area near Varna (43°12′ N, 27°55′ E), from a cultivated site.

The plant material was authenticated by a botanist at the Faculty of Biology, University of Bucharest, and voucher specimens were deposited at the university herbarium (Voucher No. RO23-DO and BG23-VR). The freshly harvested leaves were washed with distilled water to remove surface contaminants, air-dried at room temperature, and stored at 4 °C in perforated polyethylene bags for no longer than 48 h before processing. Each 10 g sample was finely chopped and placed in tightly closed vials, to which 100 mL of ethanol at varying concentrations (96%, 70%, and 40%) was added.

The maceration process was conducted over 15 days at room temperature (~20 °C) in the absence of light to preserve the integrity of the bioactive compounds. Daily shaking was performed to enhance extraction efficiency. After the maceration period, the extracts were filtered using Whatman filter paper to separate the plant material from the ethanolic solution. The resulting extract was stored in dark, airtight containers at 4 °C for further use in formulation preparation.

For this study, rosemary hydroalcoholic macerates were prepared using ethanol at 96%, 70%, and 40%, with samples from both Dobrogea (Romania) and Bulgaria. The Dobrogean rosemary macerates were labeled as RDS1 (96% ethanol), RDS2 (70% ethanol), and RDS3 (40% ethanol), while the Bulgarian rosemary macerates were designated as RBS1 (96% ethanol), RBS2 (70% ethanol), and RBS3 (40% ethanol).

For the spectrometric measurements, all the rosemary macerate samples were diluted at a 1:10 ratio before analysis. Precision and accuracy were confirmed through triplicate measurements, and reproducibility was evaluated using standard reference materials.

### 4.1. Total Polyphenol Content Analysis

Total polyphenol content (TPC) was determined using a UV-VIS Jasco V550 spectrophotometer, following the Folin–Ciocâlteu method. Absorbance was measured at 681 nm. A calibration curve using gallic acid standards (0.68 to 4.72 mg GAE/L) was established (R^2^ = 0.99923, equation: y = 0.0981x − 0.0373). The final results were expressed in mg GAE/100 g fresh weight [13,14,15,16].

### 4.2. Antioxidant Activity Analysis

Antioxidant activity was evaluated using the DPPH radical scavenging assay. The assay was performed using gallic acid as the standard, and the results were expressed in mg GAE/100 g fresh weight. Absorbance was measured at 530 nm. The calibration curve for gallic acid (0–4.76 mg GAE/L) had a correlation coefficient of R^2^ = 0.99565 (equation: y = 1.9222x − 0.3772) [13,14,15,16].

### 4.3. Total Flavonoid Content Analysis

Total flavonoid content (TFC) was determined using the aluminum chloride colorimetric method, with quercetin as the standard. The calibration curve for quercetin (1.32–7.92 mg QE/L) was linear (R^2^ = 0.99912, equation: y = 1.062x + 0.539). Results were expressed in mg QE/100 g fresh weight [12].

### 4.4. Metal Concentrations Analysis

Metal concentrations were determined using a ContrAA^®^ 700 atomic absorption spectrometer (Analytik Jena GmbH+Co. KG, Jena, Germany) with flame atomization. Samples were digested using 10 mL of concentrated nitric acid (HNO_3_) and 2 mL of hydrogen peroxide (H_2_O_2_) in a microwave digestion system. Calibration was performed using multi-element standard solutions (Certipure^®^, Merck, Darmstadt, Germany), containing magnesium, sodium, potassium, calcium, zinc, iron, copper, manganese, nickel, lead, cadmium, and chromium [14].

Table S1 from the Supplementary Materials shows the following working parameters: the concentration range (µg/L) and correlation coefficients of the calibration curve (R^2^).

### 4.5. Antimicrobial Assay

The antimicrobial activity of the rosemary macerates was tested against three reference strains: *Staphylococcus aureus* ATCC 25923 (Gram-positive cocci), *Escherichia coli* ATCC 25922 (Gram-negative bacilli), and *Candida albicans* ATCC 10231 (yeast). All strains tested were provided by the Microorganisms Collection of the Department of Microbiology, Faculty of Biology & Research Institute of the University of Bucharest.

Only the macerates RDS2 and RBS2 were selected for antimicrobial testing, as they were found to be the richest in polyphenolic compounds and exhibited the highest antioxidant activity values and antimicrobial activity in previous analyses.

Experimental Conditions: Muller–Hinton agar plates were inoculated with standardized microbial suspensions (McFarland 0.5); tested samples: rosemary macerates (RDS2 and RBS2) and an ethanol control were applied in 5, 10, 15, 20, 25, and 30 µL amounts; incubation: 18 ± 2 h at 35 ± 2 °C under aerobic conditions; and antimicrobial activity was assessed by measuring inhibition zone diameters.

Results were interpreted as sensitivity (S), resistance (R), or decrease in population density (DP) when partial inhibition was observed without clear zone formation [26].

### 4.6. Formulation of Dermatocosmetic Products with Rosemary Macerates

Preformulation conditions

The following rosemary (*Rosmarinus officinalis* L.) macerations in 70% ethanol were chosen due to their superior antioxidant activity and high total polyphenol content: RDS2 and RBS2.

Different concentrations of rosemary macerate (e.g., 0.5%, 1%, 2.5%, and 5% *w*/*w*) were tested by direct incorporation into gel and cream bases. Based on the experimental results, 2.5% *w*/*w* rosemary macerate was identified as optimal for gels prepared with 1.5% Carbopol 940. This concentration offered the following characteristics: sufficient antioxidant activity for protective effects, balanced antimicrobial efficacy without compromising skin compatibility, and good viscosity and spreadability. For creams, a slightly higher concentration, 5% *w*/*w*, may have been preferred due to the emulsion’s capacity to accommodate higher loads of active ingredients while maintaining stability and user comfort. By balancing bioactivity, stability, and sensory attributes, the chosen concentrations ensured the formulations were both effective and consumer-friendly.

Preparation

Two hydrogel formulas (Formula A and Formula B) were prepared with the composition presented in Table S2 from the Supplementary Materials. Carbopol 940, corresponding to a final concentration of 1.5%, was hydrated using purified water for at least 24 h in the presence of glycerin as a dispersing agent (purity over 99%; glycerin from Merck, Darmstadt, Germany). Triethanolamine (purity over 99%; Triethanolamine from Carl Roth GmbH + Co. KG., Karlsruhe, Germany) was used for neutralization at the end of 24 h in the cold, and added to the semisolid matrix generated by the hydration of the hydrophilic polymer under intense stirring (2000 rpm for 10 min, using a turbine stirrer in a Heidolph RZR 2020, Heidolph Instruments GmbH & Co. KG, Schwabach, Germany). Finally, rosemary macerate and the volatile oil (rosemary essential oil) were and added to the semisolid matrix under stirring until complete homogenization. The volatile oil used to prepare the gels was an organic product from Mayam, with a quality certificate (certified by Ecocert Greenlife according to the Ecocert/Cosmos standard).

Two cosmetic emulsion formulas (Formula C and Formula D) were prepared with the composition presented in Table S3 in the Supplementary Materials. The fatty components for pharmaceutical use (cetyl alcohol from Natur all Home Company, Salonta, Bihor, Romania, purified beeswax from the Mayam Company, cocoa butter bio from the Mayam Company, pure anhydrous lanolin from the Mayam Company, extra virgin sesame oil from the Mayam Company, Saint-Jean-de-Luz, France) were melted in a water bath, added in descending order of melting point until fluidization, avoiding overheating, and then mixed with extra virgin sesame oil from the Mayam Company. Separately, the purified water was heated to a temperature equal to the fluidized and homogenized fatty phase and gradually added under continuous stirring until incorporated. Finally, when the homogenized mixture had cooled to room temperature, the hydroalcoholic macerate was incorporated under continuous stirring and added gradually, followed by the rosemary essential oil. The preparations obtained were water-in-oil cosmetic emulsions, with lanolin and cetyl alcohol as emulsifiers.

### 4.7. Characterization of Dermatocosmetic Preparations

The characterization of the dermatocosmetic preparations involved several analyses, including visual examination, pH measurement, rheological behavior, and a spreadability assessment [16].

The pH of all the dermatocosmetic formulations (creams and gels) was measured using a digital pH meter (Hanna Instruments, Woonsocket, RI, USA). The pH meter was calibrated using two-point calibration:

- pH 4.00 buffer solution (for acidic formulations);
- pH 7.00 buffer solution (for neutral formulations)

The calibration process was conducted prior to each measurement to ensure accuracy. Calibration was checked after every 10 samples or whenever the pH meter was exposed to new or extreme conditions (e.g., temperature fluctuations).

Formulations were mixed thoroughly before measurement, ensuring uniform pH distribution across samples. The pH was recorded after a stabilization period of 30 s, and three readings were taken for each formulation. If the pH readings deviated by more than ±0.1 pH unit, the sample was re-measured.

Appearance analysis: Appearance was evaluated by spreading a thin layer of a sample onto a microscope slide and examining it under a magnifying glass (4.5× magnification) to observe texture and uniformity.

pH determination: The pH was measured after processing the preparations. Samples were extracted in a 1:10 ratio with distilled water, heated in a water bath at 60 °C, and homogenized for 10 min. The aqueous phase was then separated, and the pH was recorded using a multiparametric pH meter from Hanna Instruments (Hanna Instruments, Woonsocket, RI, USA).

Spreadability evaluation: The spreadability was assessed 30 h after formulation by placing 1 g of a sample between two 20 × 20 cm glass plates for 1 min, following the Ojeda–Arbussa method. A 125 g standardized upper plate was used initially, and additional weights (50 g, 100 g, 150 g, 200 g, and 250 g) were incrementally applied at 1 min intervals. The spread diameters were measured in millimeters. This process was repeated 30 and 60 days post-preparation for all samples. The results were expressed as the spread surface area in relation to the applied weight, calculated using Equation (1).

Si = di^2^ (π/4) (1)

where

- Si is the spreading area (mm^2^) resulting from the applied mass “i” (g);
- di is the mean diameter (mm) reached by the sample.

Rheological measurements

The dermatocosmetic formulations underwent detailed rheological analysis using a cylindrical system with a defined geometry. Key rheological parameters were calculated using mathematical models outlined in Table 11. The apparent viscosity, representing the viscosity at a specific shear rate, was determined through Equation (2) in Table 11, enabling the creation of viscosity curves. Rheograms for the dermatocosmetic formulations were generated using Equation (3) in Table 11, which correlated shear stress (τ) with shear rate (D). The shear rate (D, in s^−1^) was computed via Equation (4) in Table 11, based on the rotational speed (ω, in rpm). A specific constant (R) for each rotational axis was applied to derive D from ω. Additionally, shear stress (τ) was determined using Equation (5) in Table 11.

The rheological properties were assessed at varying rotational speeds (ω) between 4 and 200 rpm. The measurements were conducted with a Rotational Viscometer ST-2020 R, manufactured by Laboquimia, Spain, using 10 s intervals for each determination. Viscosity evaluations were performed with the R5 and R6 spindles, which were chosen to match the viscosity range of the samples. These spindles facilitated the calculation of the shear rate (D) in relation to the rotational speed (ω).

Regarding the comparison of the results with a known rheological model (Ostwald de Waele), Equation (6) was applied [16].

### 4.8. Determination of Antioxidant Activity

The antioxidant activity of the hydrogel formulas (Formula A and Formula B) was evaluated using the DPPH radical scavenging assay, with gallic acid (GAE) as the standard for plotting calibration curves [18]. Results were expressed in milligrams of gallic acid equivalents (mg GAE) per 100 g of fresh product, and absorbance was measured at 530 nm.

The calibration curve for gallic acid was linear across the concentration range of 0–4.76 mg GAE/L, with a correlation coefficient (R^2^) of 0.99565. The calibration curve equation was as follows: y = 0.0981x − 0.0373.

For the antioxidant activity assay, 1 mL of hydrogel diluted with 0.1 mL ethanol was mixed with 3 mL of 0.063% DPPH solution in a 25 mL volumetric flask. The solution was brought to volume with methanol and incubated in the dark for 45 min. Absorbance was then measured at 530 nm.

The total antioxidant capacity of the fat-soluble components in Preparations C and D was assessed using the photochemiluminescence method. For this analysis, 1 g of each formulation was dissolved in 10 mL of n-butyl alcohol and then filtered through paper to ensure clarity. The antioxidant capacity was measured for both the undiluted stock solution and a 1:100 dilution prepared with reagent R1 (from the ACL kit) using ethanol, following the ACL procedure (Analytik Jena AG) [16,27].

For each sample, 5 μL aliquots from the supernatant were exposed to external radiation emitted by a phosphor-coated mercury lamp, which delivers peak energy at 351 nm. This radiation, combined with a photosensitive reagent, generated superoxide anion free radicals, initiating a photochemical reaction. The antioxidants within the sample partially neutralized the radicals, and the remaining luminescence from the residual radicals was recorded as an electrical signal over 120 s. This signal was subsequently converted into concentration values.

The analysis utilized a standardized reagent kit (Analytik Jena GmbH+Co. KG, Jena, Germany) following the ACL procedure, which included R1 (dilution solvent), R^2^ (buffer solution), R3 (photosensitive reagent), and R4 (calibration reagent). The sample mixtures were prepared according to the protocol detailed in Table S4 in the Supplementary Materials.

The calibration curve was calculated using a series of standard solutions containing 0.5, 1.0, 2.0, and 3.0 nM Trolox (6-hydroxy-2,5,7,8-tetramethylchroman-2-carboxylic acid), a vitamin E derivative. Determinations were performed in triplicate and were expressed as nM Trolox equivalents (TE)/µL sample and mg (TE)/100 g d.w. sample, respectively. The correlation coefficient (R^2^) had a value of 0.9948, and the calibration curve equation was as follows: y = 1.64 x + 1.046.

### 4.9. Evaluation of Anti-Inflammatory Action of Dermatocosmetic Preparations

This preclinical study was approved by the Scientific Research Ethics Committee of the Carol Davila University of Medicine and Pharmacy, Bucharest, according to Notice 6485/12 March 2024, and complied with the regulations in force regarding preclinical testing on experimental animals. The tests were performed using two experimental methods of acute inflammation: edema induced in the rat paw with 10% kaolin suspension and with 6% dextran solution. Edema was induced by injection into the paw of 0.1 mL of 10% kaolin suspension or 0.2 mL of dextran solution [16].

A total of 6 groups of 10 male Wistar rats weighing 190 ± 15 g were used for each edematous agent. One group constituted the control group, four groups were treated with the tested preparations, Formulas A, B, C, and D, and one group was treated with Diclofenac gel 5%, produced by the company Terapia (reference group).

The volumes of the rat paws were measured plethysmometrically after the intraplantar injection of the edematous agent, and plethysmometric measurements were further performed with a plethysmometer, Ugo Basile 7140 (Gemonio, Italy), at the following intervals: 2 h, 4 h, 6 h, and 24 h (for 10% kaolin suspension the edematous agent) and 30 min, 60 min, 90 min, and 120 min from edema induction (for the 6% dextran solution edematous agent).

The mean value of anti-inflammatory edema (expressed in mL), the standard error, and the percentage of edema inhibition were calculated for each batch, according to the following formula:

Edema inhibition, % = (X control − X tested preparation/X control) × 100

where

X tested preparation represents the mean value of edema produced by the tested preparation (Formula A, B, C, D or Diclofenac gel);

X control represents the average value of edema produced in the control in the same time interval after the administration of the edematous agent.

### 4.10. Statistical Analysis

All experiments were performed in triplicate, and the results were expressed as mean ± standard deviation (SD) along with 95% confidence intervals (CI) for key measurements. A power analysis was conducted using G*Power 3.1 to determine the appropriate sample size, ensuring a significance level (α) of 0.05, power (1-β) of 0.80, and an estimated effect size (Cohen’s d) derived from pilot data. Based on this analysis, a minimum of 15 samples per ethanol concentration group was required for robust statistical comparisons.

Statistical analyses were performed using IBM SPSS Statistics 27 and GraphPad Prism 9. ANOVA was used to evaluate significant differences in total polyphenol content, flavonoid content, antioxidant activity, and mineral composition across different ethanol concentrations. Post hoc comparisons were performed using the Tukey HSD test to identify pairwise differences. Independent *t*-tests were used to compare mineral content differences between samples from Dobrogea and Bulgaria. Pearson’s correlation coefficients were calculated to assess the relationships between antioxidant activity, phenolic content, and flavonoid content, with a significance threshold set at *p* < 0.05.

## Figures and Tables

**Table 1:** Pearson correlation analysis between antioxidant activity and total polyphenolic and flavonoid content.

Correlations				
		TPC	AA	TFC
TPC	Pearson Correlation	1	0.953 **	0.842 *
	Sig. (2-tailed)		0.003	0.035
	N	6	6	6
AA	Pearson Correlation	0.953 **	1	0.937 **
	Sig. (2-tailed)	0.003		0.006
	N	6	6	6
TFC	Pearson Correlation	0.842 *	0.937 **	1
	Sig. (2-tailed)	0.035	0.006	
	N	6	6	6

* indicates correlation is significant at the 0.05 level (2-tailed). ** indicates correlation is significant at the 0.01 level (2-tailed).

**Table 2:** One-sample test.

	Test Value = 0					
	t	df	Sig. (2-Tailed)	Mean Difference	95% Confidence Interval of Difference	
					Lower	Upper
TPC	4.252	5	0.008	1019.16667	403.0361	1635.2973
AAE	3.266	5	0.022	311.25000	66.3088	556.1912
TFC	15.651	5	0.000	224.26667	187.4329	261.1004

**Table 3:** Antimicrobial activity of RDS2 and RBS2 macerates.

Organism	Volume (µL)	RDS2		RBS2		Control	
		Inhibition Zone (mm)	Sensitivity (S)/Resistance (R)	Inhibition Zone (mm)	Sensitivity (S)/ Resistance (R)	Inhibition Zone (mm)	Sensitivity (S)/ Resistance (R)
*Escherichia coli* ATCC 25922	5	↓DP	R	0	R	0	R
	10	↓DP	R	0	R	0	R
	15	11 ± 0.33	S	0	R	↓DP	R
	20	15 ± 0.55	S	↓DP	R	↓DP	R
	25	17 ± 0.27	S	↓DP	R	↓DP	R
	30	10 ± 0.46	S	↓DP	R	↓DP	R
*Staphylococcus aureus* ATCC 25923	5	11 ± 0.63	S	0	R	0	R
	10	16 ± 0.33	S	0	R	0	R
	15	19 ± 0.25	S	↓DP	R	↓DP	R
	20	21 ± 0.68	S	↓DP	R	↓DP	R
	25	23 ± 0.35	S	↓DP	R	↓DP	R
	30	17 ± 0.48	S	↓DP	R	↓DP	R
*Candida albicans* ATCC 10231	5	9 ± 0.55	R	0	R	10 ± 0.82	S
	10	11 ± 0.75	R	↓DP	R	13 ± 0.44	S
	15	14 ± 0.33	S	12 ± 0.66	S	15 ± 0.33	S
	20	15 ± 0.26	S	15 ± 0.42	S	17 ± 0.37	S
	25	17 ± 0.84	S	16 ± 0.63	S	9 ± 0.58	S
	30	20 ± 1.33	S	17 ± 0.25	S	21 ± 1.25	S

DP—decrease in population density; 0—no inhibition zone observed.

**Table 4:** Characteristics of hydrogels based on rosemary macerates.

Characteristics	Formula A	Formula B
Organoleptic evaluation—after 24 h	appearance: homogeneous, translucent; color: green; smell: specific	appearance: homogeneous, translucent; color: yellow-greenish; smell: specific
Organoleptic evaluation—after 30 days	constant initial characteristics	constant initial characteristics
Organoleptic evaluation—after 60 days	constant initial characteristics	constant initial characteristics
pH—after 24 h	4.8–5.0	4.7–4.9
pH—after 30 days	5.0–5.2	5.1–5.3
pH—after 60 days	5.4	5.5
Viscosity—after 24 h	680 ± 0.66 mPa·s	655 ± 0.25 mPa·s
Viscosity—after 30 days	662 ± 0.75 mPa·s	633 ± 0.66 mPa·s
Viscosity—after 60 days	634 ± 0.36 mPa·s	610 ± 0.52 mPa·s

**Table 5:** Characteristics of dermatocosmetic creams based on rosemary macerates.

Characteristics	Formula C	Formula D
Organoleptic evaluation—after 24 h	appearance: homogeneous; color: greenish; smell: specific	appearance: homogeneous; color: yellow-greenish; smell: specific
Organoleptic evaluation—after 30 days	constant initial characteristics	constant initial characteristics
Organoleptic evaluation—after 60 days	constant initial characteristics	constant initial characteristics
pH—after 24 h	5.0–5.2	5.3–5.4
pH—after 30 days	5.2–5.4	5.4–5.5
pH—after 60 days	5.5	5.6
Viscosity—after 24 h	978 ± 0.36 Pa·s	956 ± 0.25 Pa·s
Viscosity—after 30 days	956 ± 0.45 Pa·s	923 ± 0.55 Pa·s
Viscosity—after 60 days	930 ± 0.33 Pa·s	902 ± 0.66 Pa·s

**Table 6:** Value intervals for rheological parameters.

Sample	Shear Spead D (s^−1^) Interval	Viscosity ƞ (cP) Interval	Shear Stress τ (mPa) Interval
Formula A	2.14–62	2100–32,198	64,500–150,180
Formula B	3.2–62	619–46,212	42,400–152,720
Formula C	2.12–34	32,800–106,100	115,202–341,900
Formula D	2.04–32	8400–101,900	143,016–369,600

**Table 7:** The coefficient values of the Ostwald de Waele rheological model.

Sample	K Consistency Coefficinet	n Flow Coefficient	R Correlation Coefficient, Ostwald de Waele
Formula A	12.841	0.4335	0.9973
Formula B	11.388	0.3687	0.9982
Formula C	11.544	0.7033	0.9974
Formula D	11.632	0.4014	0.9984

**Table 8:** The regression analysis. The dependent variable pH was modeled according to the predictor “Viscosity”.

Model	R	R Square	Adjusted R Square	Std. Error of Estimate	Change Statistics					Durbin–Watson
					R Square Change	F Change	df1	df2	Sig. F Change	
1	0.320 ^a^	0.102	0.013	0.28881	0.102	1.139	1	10	0.311	2.200
a. Predictors: (Constant), Viscosity										

**Table 9:** Regression coefficients for expressing viscosity as function of pH.

Coefficients						
Model		Unstandardized Coefficients		Standardized Coefficients	t	Sig.
		B	Std. Error	Beta		
1	(Constant)	4.736	0.450		10.522	0.000
	Viscosity	0.001	0.001	0.320	1.067	0.311

**Table 10:** Antioxidant activity of dermatocosmetic preparations.

No	Sample/Dilution/Working Volume	Free Radical Max. Inhibition	Total Antioxidant Capacity (nM TE/μL)	*TEAC* Quantity Means (mg TE/100 g Sample)
1.	Formula A/stock sol./5 μL;	0.965	−3.504	-
2.	Formula B/stock sol./5 μL;	0.645	−2.304	-
3.	Formula C/stock sol./5 μL;	0.874	−3.849	-
4.	Formula D/stock sol./5 μL;	0.704	−2.756	-

**Table 11:** Equations used in rheological measurements.

η = f(D) (2)	D = f(τ) (3)	D = ω R (4)	τ = η D (5)	*τ* = *k* *D*^*n*^ (6)

## Data Availability

The original contributions presented in the study are included in the article, further inquiries can be directed to the corresponding author.

## Supplementary Materials

The following supporting information can be downloaded at: https://www.mdpi.com/article/10.3390/gels11030149/s1, Table S1: Performance parameters for AAS measurements.; Table S2: Composition of hydrogels with rosemary macerated in 70% ethanol.; Table S3: Composition of cosmetic emulsions with rosemary macerated in 70% ethanol.; Table S4: Working scheme of ACL procedure.
