# Supplementary material for: The Development and Comparative Evaluation of Rosemary Hydroalcoholic Macerate-Based Dermatocosmetic Preparations: A Study on Antioxidant, Antimicrobial, and Anti-Inflammatory Properties
Source: Gels. 2025 Feb 20;11(3):149. doi: 10.3390/gels11030149 (PMC11942321; doi:10.3390/gels11030149)
Supplement: Supplementary file 1 [file gels-11-00149-s001.zip › gels-3461375-supplementary.pdf]

Table S1. Performance parameters for AAS measurements.

Metal	Concentration domain (mg/L)	R^2	Calibration curve equation
Cadmium	0.00040 – 0.0040	0.9989	y = 0.0122973x + 0.0422208
Calcium	40.00 – 200.00	0.9996	y = 0.000138x + 0.0000701
Chromium	0.002 – 0.020	0.9913	y = 0.0138310x + 0.0125257
Copper	0.003 – 0.030	0.9958	y = 0.1937482x + 0.0141176
Iron	0.05 – 2.00	0.9929	y = 0.0007637x + 0.0308354
Lead	0.01 – 0.10	0.9976	y = 0.0145906x+ 0.0042433
Magnesium	1.0 – 5.0	0.9932	y = 0.0062880x +0.0571131
Manganese	0.0015 – 0.0150	0.9950	y = 0.0129916x + 0.0202151
Nickel	0.007 – 0.070	0.9926	y = 0.0107338 x + 0.0041630
Potassium	1.0 – 5.0	0.9975	y = 0.0013933x + 0.0024000
Sodium	5.0 – 25.0	0.9966	y = 0.0000076x + 0.0034889
Zinc	0.0005 – 0.005	0.9925	y = 0.3079303x + 0.0533993

Table S2. Composition of hydrogels with Rosemary macerated in 70% ethanol.

Components	Mass (g)	
	Formula A	Formula B
Carbopol 940	1.5 g	1.5 g
Glycerine	5 g	5 g
Triethanolamine	q.s	q.s
Rosemary essential oil	1 g	1 g
RDS2	2.5 g	-
RBS2	-	2.5 g
Purified water	until 100 g	until 100 g

q.s - quantum satis

Table S3. Composition of cosmetic emulsions with Rosemary macerated in 70% ethanol.

**Components**	**Mass (g)**	
	**Formula C**	**Formula D**
Cetyl alcohol	1.5 g	1.5 g
Beeswax	4 g	4 g
Cocoa butter	10 g	10 g
Lanolin anhydrous	2 g	2 g
Sesame oil	32 g	32 g
Rosemary essential oil	2 g	2 g
RDS2	5 g	-
RBS2	-	5 g
Purified water	until 100 g	until 100 g

Table S4. Working scheme of ACL procedure.

**Reagents kit**	**R1 (µL)**	**R2 (µL)**	**R3 (µL)**	**R4 (µL)**	**Sample (µL)**
**Blank**	2300 µL	200 µL	25 µL	0 µL	0 µL
**Calibration curve**	2300 µL	200 µL	25 µL	5 µL	0 µL
**Measurement Sample**	2300 µL	200 µL	25 µL	0 µL	5 µL
